# Impact of Retinal Degeneration on Response of ON and OFF Cone Bipolar Cells to Electrical Stimulation

**DOI:** 10.1109/TNSRE.2023.3276431

**Published:** 2023-05-26

**Authors:** Shayan Farzad, Pragya Kosta, Ege Iseri, Steven T. Walston, Jean-Marie C. Bouteiller, Rebecca L. Pfeiffer, Crystal L. Sigulinsky, Jia-Hui Yang, Jessica C. Garcia, James R. Anderson, Bryan W. Jones, Gianluca Lazzi

**Affiliations:** Department of Biomedical Engineering, University of Southern California, Los Angeles, CA 90033 USA; Institute for Technology and Medical Systems (ITEMS), Keck School of Medicine, University of Southern California, Los Angeles, CA 90089 USA.; Department of Biomedical Engineering, University of Southern California, Los Angeles, CA 90033 USA; Institute for Technology and Medical Systems (ITEMS), Keck School of Medicine, University of Southern California, Los Angeles, CA 90089 USA.; Department of Biomedical Engineering, University of Southern California, Los Angeles, CA 90033 USA; John Moran Eye Center, University of Utah, Salt Lake City, UT 84132 USA.; John Moran Eye Center, University of Utah, Salt Lake City, UT 84132 USA.; John Moran Eye Center, University of Utah, Salt Lake City, UT 84132 USA.; John Moran Eye Center, University of Utah, Salt Lake City, UT 84132 USA.; John Moran Eye Center, University of Utah, Salt Lake City, UT 84132 USA.; John Moran Eye Center, University of Utah, Salt Lake City, UT 84132 USA.; Department of Electrical Engineering, Biomedical Engineering, and Ophthalmology, University of Southern California, Los Angeles, CA 90089 USA

**Keywords:** Cone bipolar cells (CBCs), computational modeling, calcium channel, retinal degeneration

## Abstract

In retinal degenerative diseases, such as retinitis pigmentosa (RP) and age-related macular degeneration (AMD), the photoreceptors become stressed and start to degenerate in the early stages of the disease. Retinal prosthetic devices have been developed to restore vision in patients by applying electrical stimulation to the surviving retinal cells. However, these devices provide limited visual perception as the therapeutic interventions are generally considered in the later stages of the disease when only inner retinal layer cells are left. A potential treatment option for retinal degenerative diseases in the early stages can be stimulating bipolar cells, which receive presynaptic signals from photoreceptors. In this work, we constructed computational models of healthy and degenerated (both ON and OFF-type) cone bipolar cells (CBCs) with realistic morphologies extracted from connectomes of the healthy and early-stage degenerated rabbit retina. We examined these cells’ membrane potential and axon terminal calcium current differences when subjected to electrical stimulation. In addition, we investigated how differently healthy and degenerated cells behave with respect to various stimulation parameters, including pulse duration and cells’ distance from the stimulating electrode. The results suggested that regardless of the position of the OFF CBCs in the retina model, there is not a significant difference between the membrane potential of healthy and degenerate cells when electrically stimulated. However, the healthy ON CBC axon terminal membrane potential rising time-constant is shorter (0.29 ± 0.03 ms) than the degenerated cells (0.8 ± 0.07 ms). Moreover, the ionic calcium channels at the axon terminals of the cells have a higher concentration and higher current in degenerated cells (32.24 ± 6.12 pA) than the healthy cells (13.64 ± 2.88 pA) independently of the cell’s position.

## I. INTRODUCTION

VISUAL perception is made possible by photon capture from photoreceptors, the sensory components of the retina, followed by algorithmic processing by downstream neurons that process visual primitives before sending the outputs to areas of the cortex and sub-cortex. In retinal degenerative diseases such as retinitis pigmentosa (RP) and age-related macular degeneration (AMD), the photoreceptors become stressed and begin to degenerate in the early stages of the disease. Later stages of the disease then lead to complete loss of vision. It has been shown that the absence of presynaptic signals from the photoreceptors in retinal degeneration results in extensive restructuring of the neural circuitry, gliosis, and morphological alterations in the remaining layers of the retina [1], [2], [3], [4]. Additionally, even prior to photoreceptor cell loss, and the presumed loss of signaling, retinal plasticity ensues [2], [5], [6], altering the network topology and glutamate receptor pharmacology of retinal circuits.

Retinal prosthetics have been developed to help individuals with retinal degenerative diseases achieve a perception of objects, letters, and colors by functioning as photoreceptor surrogates, electrically stimulating the remaining retinal neurons [7], [8], [9], [10], [11], [12]. In the epiretinal prostheses, the primary stimulation targets are ganglion cells (GCs), the output layer of retinal networks, sending the signals to the brain [13], [14]. Overall, two types of neural activation are targeted in epiretinal stimulation: direct and indirect activation of GCs. Both stimulation strategies have their challenges and limitations: (1) direct activation results in axonal activation of the target GCs near the stimulating electrode and neighboring GCs, which leads to the appearance of extended phosphene and low spatial resolution of the devices [14], [15]. Further, a higher stimulation current is needed to activate degenerated GCs compared to healthy GCs [16], potentially contributing to tissue damage or restructuring. (2) Indirect activation of GCs, on the other hand, is done via activation of bipolar cells (BCs). A previous study suggested that the stimulation thresholds of BCs do not differ significantly between the healthy and degenerate retina [17]. However, efficient indirect activation of GCs depends on the stage of degeneration and inherently relies on the surviving retinal network. Even in the early stages of degeneration, aberrant wiring (including the appearance of novel gap junctions) is found within the retinal network [18], [19]. Additionally, altered glutamate channel activity in BCs early in retinal degeneration likely results in altered BC function [2], [5], [6]. The impact of this retinal plasticity, including the loss of retinal cells and alterations of both chemical and electrical synapses, on retinal processing is not well understood and is an active area of investigation. Computational modeling is required to understand how current flows in the healthy and degenerate retinal networks through the simulation of the altered retinal networks, the impact of lost/new synapses, and will ultimately provide a resource for the design of stimulation strategies that are effective in activating the degenerated retinal network.

In *in-vitro* investigations, the response of retinal neurons to electrical stimulation primarily focused on GCs as the retina’s output neurons. However, BCs also play a critical role in visual processing by initially segregating the signals into ON and OFF components as they transmit information from photoreceptors to inner-layer GCs [20]. Amacrine cells further modify these signals at the level of the BC axon terminals, in addition to direct input to GC dendrites. Consequently, alongside the extensive advancement of GC models, several electrophysiological studies have focused on understanding the behavior of BCs by characterizing the functionality of their voltage-gated ion channels [20], [21]. Additionally, a limited number of experimental studies have precisely measured the BC activation in response to electrical stimulation of degenerate retina [17], [21], [22], [23]. Thorough investigations with more complete networks have yet to be completed.

Some computational studies have investigated single-compartment conductance-based models for simulating the biophysics of different retinal cells. They use the electrical circuits to understand the behavior of different classes of retinal cells relative to the stimulating electrodes and reproduce the results obtained from electrophysiological recordings of the retina [24], [25]. Although these modeling approaches yield the interpretation of different classes of retinal cells, most of these studies lack morphological realism. In these single-compartment modeling approaches, neurons are treated as isoelectric. We now know that the accurate representation of the neurons requires multi-compartment modeling. Maturana et al. [26] have investigated the effect of cell morphology on intrinsic electrophysiological properties by using a multicompartmental model of ON and OFF retinal GCs. A more recent study [27] analyzes the impact of the number of model compartments on simulation accuracy for spiking bipolar cells. The study suggests that accurate modeling of extracellular stimulation demands many short compartments even for passive membranes. Recently, a model-based spiking bipolar cell model has been developed in our group, and the ion channel distribution has been evaluated and compared with mice experimental data [17], [28]. Our group has also developed models that represent the architecture of healthy and degenerative retinas with appropriate resistivity for each layer of the retina (and adjusted thickness for the degenerated retina) [16]. Although the model was synthetically adjusted to resemble the behavior of degenerated retina, the cell morphologies and synaptic connections were based on the precise retinal morphologies of both healthy and degenerate retina from ultrastructural reconstructions, giving these approaches a distinct advantage in understanding how current flows through biologically relevant retinal networks. Moreover, our group has previously performed a model-based study for rod bipolar cells to assess the effect of current flow in the healthy and early-stage degenerated retina [29]. We simulated the current flow from rod photoreceptors to rod bipolar cells (RodBCs) for both healthy and degenerated retinas. Our simulation results suggested that the functional behavior of degenerated RodBCs, in the earliest stages of retinal degeneration, is not significantly different from those in the healthy RodBCs with similar photocurrent inputs. However, RodBCs in early-stage degenerated retina receive input from fewer numbers of rod photoreceptors in comparison with healthy retina; therefore, RodBCs of the degenerated retina show lower membrane potentials than those of the healthy retina. Electrophysiological studies have shown that voltage-gated ion channels vary in different regions of BCs, yet most of the existing cone BC (CBC) models are assumed to be passive or only contain calcium channels at the axon terminals [24], [27], [30], [31], [32] Therefore, we initiated the development of realistic models of the retina, beginning with retinal BCs, to replicate the subtleties of the cells’ behavior more faithfully, enabling more sophisticated explorations of the parameters affecting the cells’ response to electrical stimulation. These models provide valuable insights to help refine stimulation strategies and improve the performance of current epiretinal prosthetic devices. Finally, more realistic BC models can help identify the mechanisms underlying network-level behavior and better understand the differences between direct and indirect activation of GCs in response to epiretinal electrical stimulation.

In this work, we have employed our multi-scale computational three-dimensional (3D) Admittance method (AM)-NEURON platform to model electrical stimulation of retinal tissue and simulate responses of ON and OFF CBCs. We construct the CBC models based on the morphology extracted from healthy and degenerated ultrastructural retinal connectomes captured at 2.18nm/pixel [11], [16], [28], [29], [33], [34], [35], [36], [37], [38], [39], [40]. Leveraging this multi-scale computational model, we compute the membrane potentials generated in different regions of healthy cells and compare them with early-stage degenerated ON and OFF CBCs in response to electrical stimulation. Further, we investigate the changes in calcium current in the synapses of the healthy and early-stage degenerated ON and OFF CBCs to determine the response to epiretinal electrical stimulation.

## II. Materials AND Methods

### A. Retinal Connectomics and Data Extraction

The connectome of a healthy retina (RC1) [41], [42] and a pathoconnectome (RPC1) of an early-stage degenerated retina from a ten-month-old transgenic (Tg) P347L rabbit RP retina model have been previously described [18]. Briefly, the connectome and pathoconnectome were built by slicing the rabbit retina tissue in 70nm-thick slices and imaging with high-resolution transmission electron microscopy (TEM) imaging approaches with a diameter of 250μm and 70μm for RC1 and RPC1, respectively. Datasets were manually annotated, identifying their cellular and subcellular components (such as pre-and postsynaptic densities, ribbon synapses, and gap junctions). Finally, 3D morphologies of retinal cell classes were created by rendering annotations with visualization tools [43], [44].

**Fig. 1.**
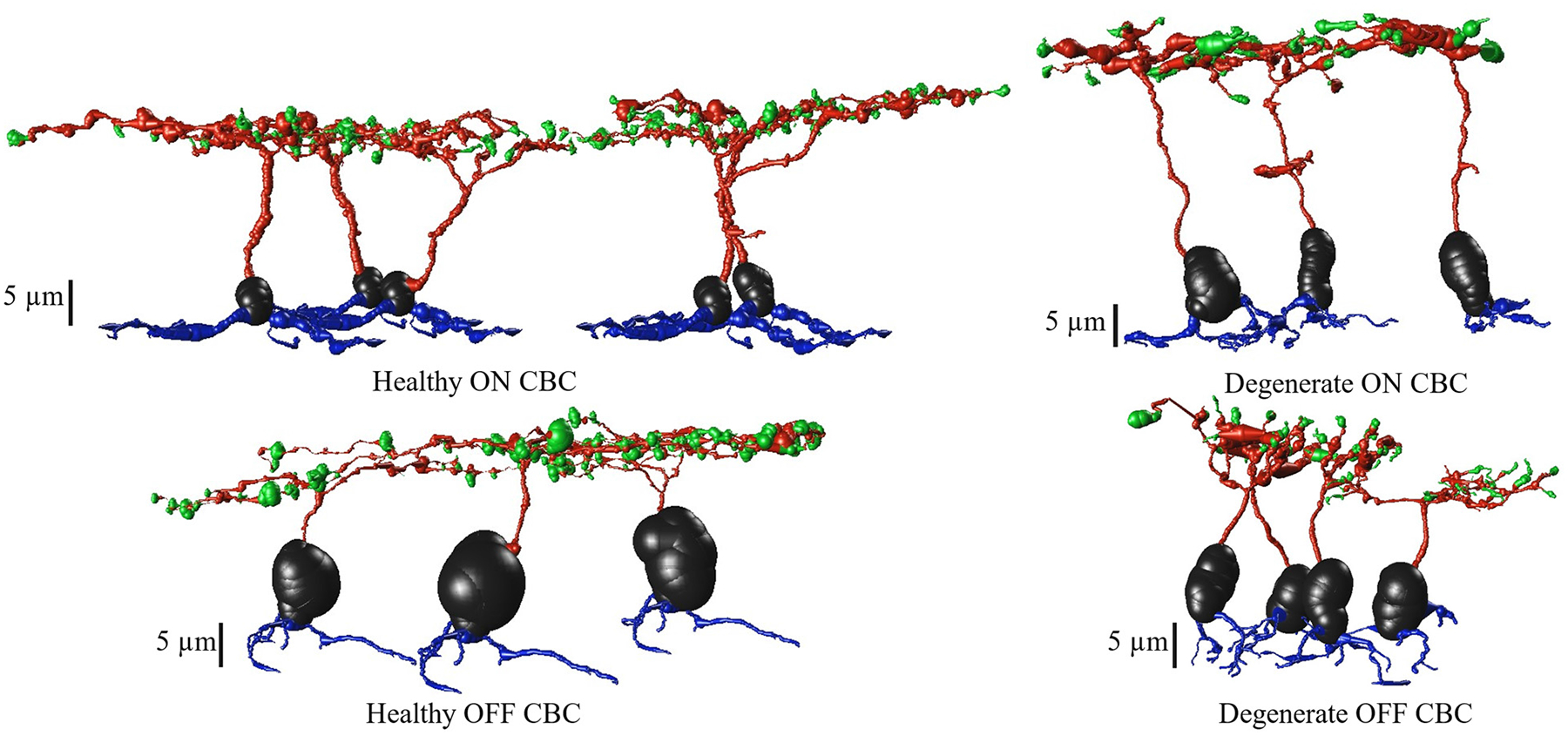
Morphology of healthy and degenerate CBCs, extracted from RC1 and RPC1, respectively. Cell somas are depicted in black, dendrites in blue, axons in red and axon terminals are in green color.

The connectome databases provide the most accurate and realistic cell morphologies, synapse identification, localization, quantification, and network topology details that provide circuit-level information. Despite this, the connectomes that are most accurate to the biological system are currently manually annotated. This is a time-consuming process involving an iterative approach that results in evolving data, meaning with additional annotation, synapse numbers and morphology may be further refined. Different classes of bipolar cells, amacrine cells, and ganglion cells, including their synaptic connections between the cells, are included in the datasets. In this manuscript, we focused on computational modeling and comparison of ON and OFF CBCs of RC1 and RPC1 to understand initial morphological changes that might impact the possibility of developing early treatment strategies preceding significant loss of the photoreceptor and bipolar cells. To build the computational model, we extracted the morphology and synaptology data of CBCs from RC1 and RPC1 connectomes. The tools used to extract and visualize the data in this project were custom Python scripts, as well as Tulip [44] and vaa3D software [45]. For these analyses, three annotated RPC1 ON CBCs (Cell IDs 430, 740 and 1167) were compared against five RC1 ON CBCs (Cell IDs 180, 419, 6156, 9693 and 16026), matched for CBC class (all CBb6) and annotation completeness. Similarly, four annotated RPC1 OFF CBCs (Cell IDs 1, 80, 352 and 1134) were compared against three RC1 OFF CBCs (Cell IDs 165, 6128 and 32359), matched for annotation completeness but not class identity. Fig. 1 presents the morphology of these cells with retinal bipolar cell dendrites represented in blue at the bottom of the figures and the axon terminals, axonal arbors of the bipolar cells at the top. The dendrites for the RC1 ON and OFF CBCs could not be directly extracted from the RC1 connectome, as this volume does not include the outer plexiform layer. Therefore, in order to have complete cells for the subsequent simulations, the dendritic arbor of a single corresponding ON (Cell 1167) or OFF (Cell 80) CBC from RPC1 (chosen for their median dendritic arbor features) was used to patch the missing part of morphology of ON and OFF RC1 CBCs, respectively.

### B. NEURON Stimulation

The alteration in cell membrane potential to an applied external stimulation is measured using NEURON computational software [46]. The extracted cells from the RC1 and RPC1 connectomes are generated in a multi-compartmental approach, where soma, axon, dendrite, and axon terminals are characterized as separate compartments [18], [41], [42].

The cells have unique biophysical properties with passive and active ionic membrane channels. The healthy and degenerate ON and OFF CBCs calcium channels have been adjusted at their axon terminals derived from the previous study of rat CBC, respectively [32]. Benison et al. [47] and Avery and Johnston [48] initially developed the activation and inactivation gates from the L-type *Ca*^2+^ currents based on a model of cat retinal ganglion cell and rat CA3 pyramidal neurons in the hippocampus. Moreover, a previously developed model for T-type *Ca*^2+^ currents in HEK-293 cells expressing human Cav3.3 channels [49] was adapted for simulating T-type *Ca*^2+^ currents in rat CBCs, featuring one activation gate and one inactivation gate.

To the best of our knowledge, there is no experimental data specifically on extracellular electrical stimulation of ON and OFF CBCs. Nonetheless, there exists a whole-cell patch clamp study that measured the L-type calcium current in the rod bipolar cell of the rat retina [50], [51], [52]. Additionally, there are experimental reports that use whole-cell patch clamp techniques to examine the T-type calcium current of the rat retinal cone bipolar cells [53], [54]. In the present study, we assume that there is a linear leak current present in all cell compartments, with only calcium L-type and T-type channels present at the synaptic terminals of ON and OFF CBCs, respectively. This assumption is based on the Werginz et al. [32] model for rat ON and OFF CBCs. We have repeated the validation part of the rat CBC model for good measure and confirmed that the behavior of the inward calcium current aligns with the experimental results [50], [51], [52], [53], [54], [55], [56]. Additionally, a comparable pattern was noted in both ON and OFF retinal ganglion cells. The conductance of T-type calcium current is much higher in OFF RGCs than in ON RGCs [57]. As there is no evidence of ionic channels specific to CBb6 of rabbit CBCs, we have adjusted Werginz et al. [32] model as a preliminary model to study morphologically realistic cells.

The ionic kinetics of L-type calcium channel of ON CBCs are:

(1) $$i_{CaL}=g_{CaL}m^{2}h\left(V-E_{CaL}\right)$$

(2) $$\frac{dm}{dt}=\alpha_{m}(1-m)-\beta_{m}m\;\frac{dh}{dt}=\frac{h_{\infty }-h}{\tau_{h}}$$

(3) $$\alpha_{m}=0.427\frac{V-63}{1-{\text{e}}^{-(V-63)/10.5}}$$

(4) $$\beta_{m}=0.0406{\text{e}}^{70-V/12}$$

(5) $$h_{\infty }=\frac{1}{1+e^{V/66.4}}and\;\tau_{h}=292ms$$

The ionic kinetics of T-type calcium channel of OFF CBCs are:

(6) $$i_{CaT}=g_{CaT}mh\left(V-E_{CaT}\right)$$

(7) $$\frac{dm}{dt}=\frac{m_{\infty }-m}{\tau_{m}}\;\frac{dh}{dt}=\frac{h_{\infty }-h}{\tau_{h}}$$

(8) $$m_{\infty }=\frac{1}{1+e^{-\frac{V-37.55}{3.07}}}\;and\;\tau_{m}=1.36+\frac{21.68}{1+e^{(V-39.96)/4.11}}ms$$

(9) $$h_{\infty }=\frac{1}{1+e^{\frac{V-8.97}{8.42}}}\;and\;\tau_{h}=65.82+0.0023e^{(V-80)/4.78}ms$$

The *Ca*^2+^ membrane conductance value for both healthy and early-stage degenerated ON and OFF CBCs has been adjusted to 0.1 mS/cm2 to achieve a similar current density response of CBCs with a higher number of axonal terminals compared with the model presented in Werginz et al. [32]. The reversal potential of the calcium channel (ECaL) is devised based on the intracellular concentration of the calcium, according to Fohlmeister and Miller [58]. The extracellular calcium concentration is set to 1.8 mM for both ON and OFF CBCs. The calcium pump’s depth and the calcium current’s time constant are 0.5 μm and 1.5 ms, respectively. The membrane capacitance and intracellular resistivity are set to 1 μF/cm2 and 100 Ώ.cm. The resting membrane voltage is −53 mV.

### C. AM-NEURON Computational Platform

**Fig. 2.**
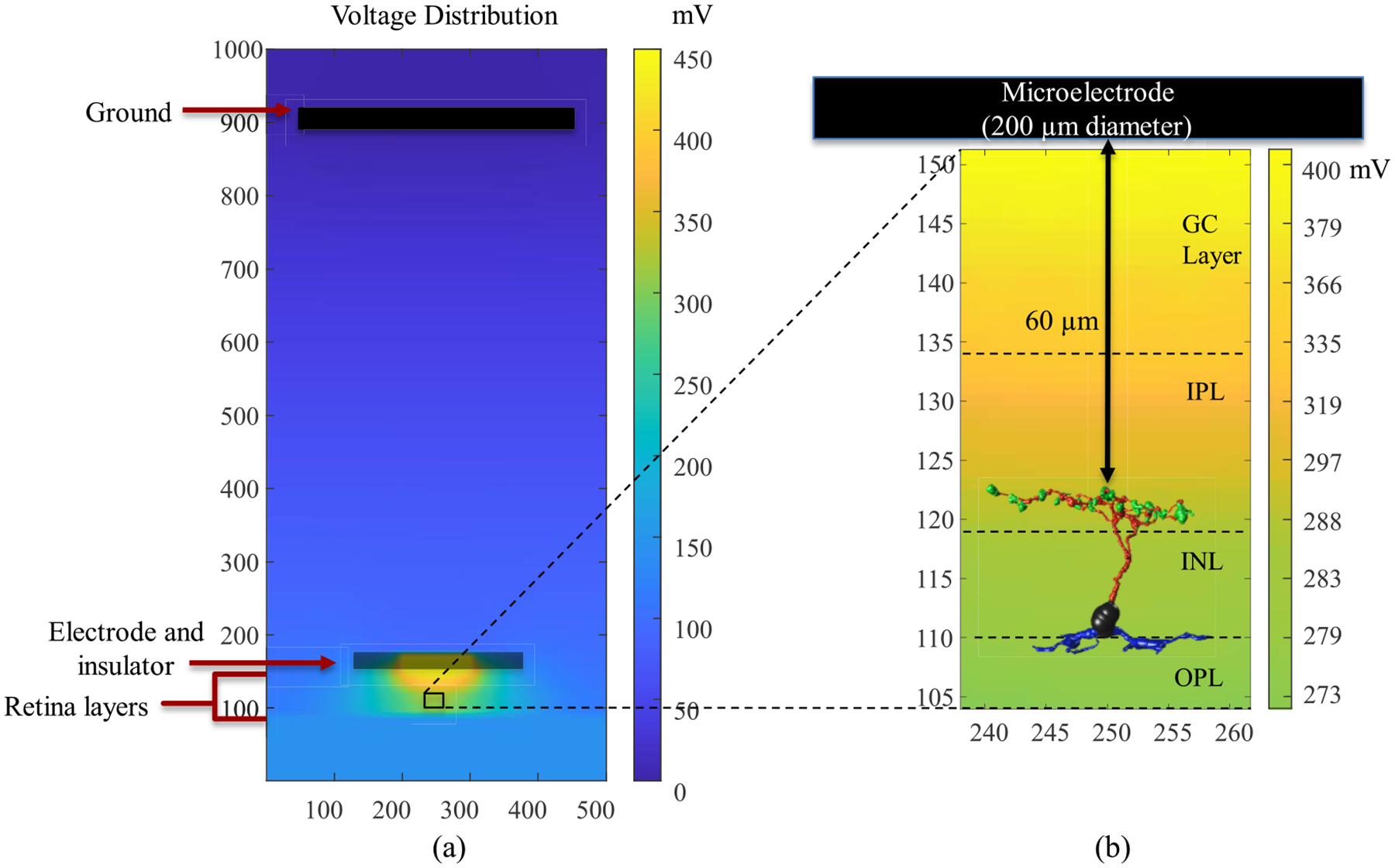
A multi-scale model consisting of (a) bulk tissue model with microelectrode, various retinal layers (GC: ganglion cell; IPL: inner plexiform layer; INL: inner nuclear layer; OPL: outer plexiform layer, ONL: outer nuclear layer and PR: photoreceptor) and (b) morphologically detailed retinal bipolar cell extracted from the connectome inside the AM-NEURON model. The stimulating electrode of 200 μm diameter is placed 60 μm from the axon terminals of BCs. The bulk retinal tissue model is utilized to compute the voltages at every node of the model due to the stimulating microelectrode. These extracellular voltages are then applied to the bipolar cell model to simulate its spatiotemporal response to electrical stimulation.

We developed a multi-scale computational model consisting of a bulk retinal tissue model and retinal cell models based on connectome databases, as shown in Fig. 2.The bulk retinal tissue model contains various retinal layers of different impedance properties and the implanted electrode [38]. Both the material conductivity and permittivity can be accounted for during the computation of voltage. However, it was observed that including relative permittivity does not affect the change in membrane potential in a meaningful way because the time resolution of milliseconds is large with respect to the time constant of the equivalent impedance. Therefore, to reduce the computational complexity of the model, the impedance values used for the retinal tissues are purely resistive.

The Admittance Method (AM) applies a mesh grid to the bulk tissue model and is used to compute the extracellular voltages induced at various nodes of the retinal tissue model. By placing the cell in the model and applying interpolation along the cell morphology, we can measure the voltage values at every center of each compartment of the retinal cells. Finally, the neuronal response is simulated by applying the interpolated voltages as the extracellular voltage at every compartment. More extensive details about the modeling approach and properties of the retinal layers can be found in previous works by our group [11], [16], [28], [29], [33], [34], [35], [36], [37], [38], [39], [40]. Because the characterization of biophysical properties between healthy and degenerating retinal cells is limited in the literature, the same conductivity values were used in both healthy and early-stage degenerated cells.

The thickness of the retina layers changes with degeneration and the cells may move within the INL layer. Additionally, RC1 was constructed from tissue located in the midperipheral retina, while tissue for RPC1 was close to the visual streak and thicker. Therefore, at first, the soma of all ON and OFF CBCs were fixated in the INL Layer, and we investigated the membrane potential response at all sections of the cell and the calcium current response at the axon terminals. We then looked at the effect of cell position in the model with respect to the stimulating electrode in healthy and degenerate cells for ON and OFF CBCs. We considered the cell with the longest axonal length, which had the closest axon terminals to the electrode, as the reference synaptic distance. Next, we positioned the remaining cells’ axon terminals at the same distance from the electrode and computed the changes in the membrane potential response at all cell sections and the calcium current response based on the new adjustment.

### D. Stimulation Pattern

**Fig. 3.**
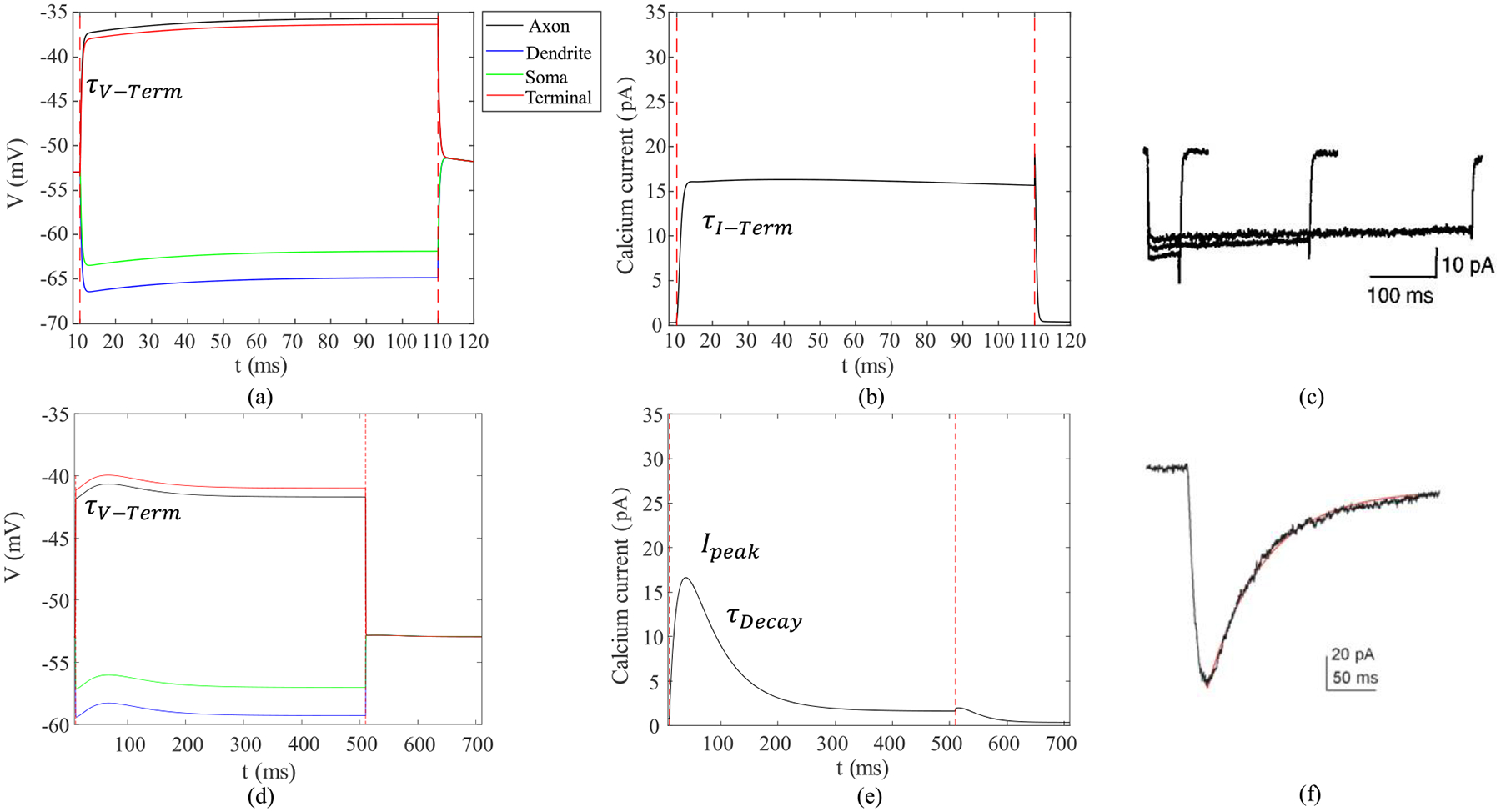
The average membrane potential and absolute inward calcium current response of all the compartments of a healthy retinal (a, b) ON CBC with pulse duration of 100ms and (d, e) OFF CBC with pulse duration of 500ms to extracellular current stimulation of 100 uA. (c) L-type calcium current response of whole-cell voltage clamp in rat retinal RBC [52]. (f) T-type calcium current response of whole-cell patch clamp in rat retinal cone bipolar cell [53]. *τ*_*V*_ − *Term* and *τ*_*I*_ − *Term* are the measured rising time constants of axon terminal membrane potential and L-type calcium current, respectively. *τ*_*De*_*cay* and *I*_*pe*_*ak* are the peak and decay time constant of T-type calcium current.

In this work, a cathodic monophasic pulse with a duration of 0.5, 1, 4, 8, 16, 25, 50, and 100 ms at 100 uA was applied on healthy and early-stage degenerate ON CBCs. The minimum pulse width of 0.5 ms confers to the Argus II implant’s pulse [59], and the upper limit was chosen following a study that showed pulse widths of up to 100 ms let to activation of inner retinal neurons such as bipolar cells while avoiding activation of ganglion cell axons [15]. As the T-type calcium channel has a longer decay duration, for OFF CBCs, we have applied identical pulses, including an additional pulse duration of 500 ms at 100 uA, to capture the complete calcium current response to the stimulation. Fig. 3 illustrates an example of the membrane potential and calcium current response of the healthy ON CBC with a pulse duration of 100ms and OFF-CBC with a pulse duration of 500ms to extracellular current stimulation. Several results in the rest of the paper are derived from the analysis of these curves. *τ*_*V*_–*T erm* and *τ*_*I*_–*Term* are the measured rising time constants of axon terminal membrane potential and L-type calcium current, respectively. *τDecay* and *I*_*peak*_ are the peak and decay time constant of T-type calcium current.

## III. RESULT

### A. Morphological Differences

**Fig. 4.**
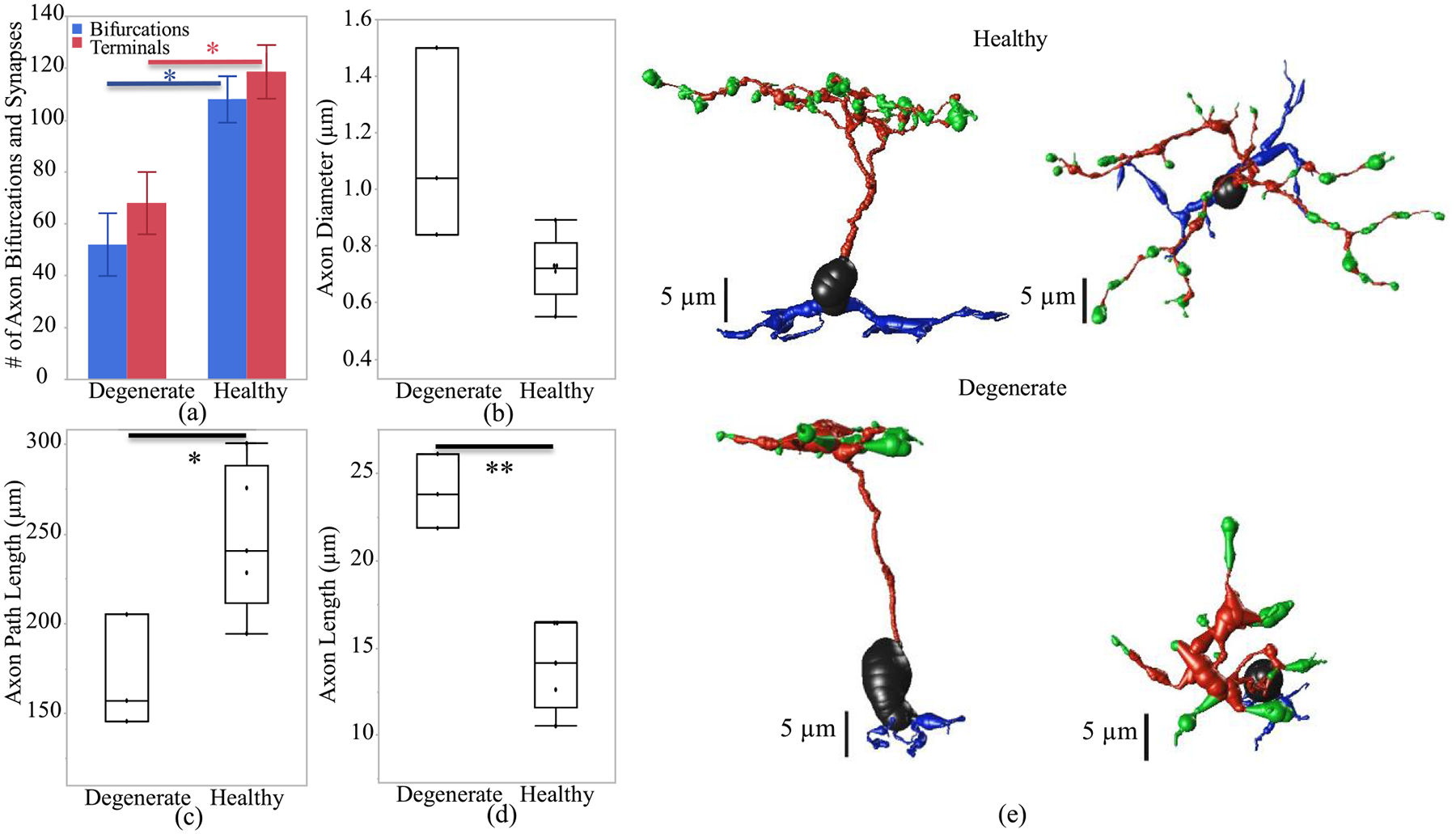
Axon morphological differences of healthy and early-stage degenerated ON CBCs. (a) Number of bifurcations and terminals of the axon, (b) diameter of the axon, (c) path length defined as the total length of the axon, (d) the axon length represents the longitudinal length of the axon before the first bifurcation point and (e) an example of ON-CBC topology from the side and top view. The * and ** are the t-test statistically significance level of ≤0.05 and ≤0.01, respectively.

**Fig. 5.**
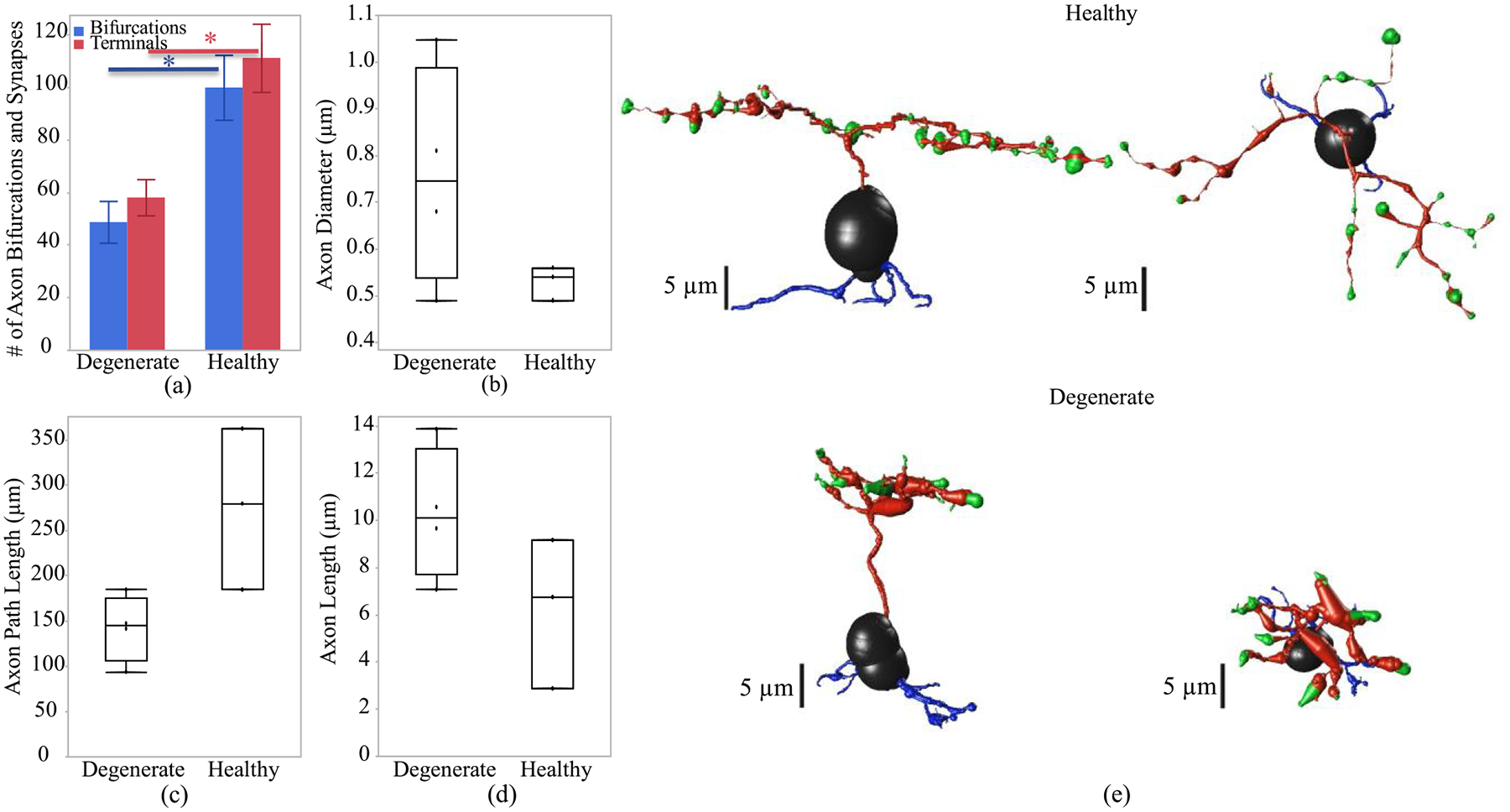
Axon morphological differences of healthy and early-stage degenerated OFF CBCs. (a) Number of bifurcations and terminals, (b) diameter of the axon, (c) path length defined as the total length of the axon, (d) the axon length represents the longitudinal length of the axon before the first bifurcation point and (e) an example of an OFF-CBC topology from the side and top view. The * is the t-test statistically significance level of ≤0.05.

To better understand the membrane potential and calcium current response at the axon terminal of the ON and OFF CBCs, we investigated the morphological differences in CBC axons between RC1 and RPC1 connectomes. Bifurcations and terminals represent the number of branches and connection points to synapses at the axon, respectively. In this analysis, the measurements are reported with mean and standard error values. The results (Fig. 4a and Fig. 5a) show that both healthy RC1 ON and OFF CBCs have a higher number of bifurcation (108 ± 9 and 100 ± 12, respectively) and terminals (119 ± 10 and 112 ± 13, respectively) compared with degenerate RPC1 cells bifurcation (52 ± 12 and 49 ± 8, respectively) and terminals (68 ± 12 and 58 ± 7, respectively) with a t-test significance level of p < 0.05. The axon diameter for healthy RC1 and degenerate RPC1 ON CBC have ranges of 0.2–1.1 μm and 0.3–1.6 μm, respectively. Also, the axon diameter for healthy RC1 and degenerate RPC1 OFF CBC have ranges of 0.1–0.8 μm and 0.3–1.2 μm, respectively. There is no significant difference in average axon diameter between healthy RC1 and degenerate RPC1 ON and OFF CBCs (Fig. 4b and 5b). Moreover, the total length of the axon, including the path along the bifurcations up to the axon terminals of the cells, i.e. path length (Fig. 4c), is significantly higher in healthy ON CBCs than in degenerated cells (247.9 ± 18.5 μm and 169.3 ± 18.3 μm, respectively). However, this difference is not significant in the healthy OFF CBCs compared with early-stage degenerated cells (Fig. 5c). Axon length (Fig. 4d) is the longitudinal length of the axon before the initial bifurcation point, and it is significantly higher in degenerate ON CBCs than the healthy cells (23.9 ± 1.2 μm and 14.0 ± 1.1 μm, respectively). However, axonal length is not substantially different for healthy OFF CBCs and early-stage degenerated OFF CBCs (Fig. 5d). An example of the longitudinal and transverse extent of a single healthy and degenerate ON CBC is presented in Figures 4e and 5e.

### B. Response to the Extracellular Current Input

**Fig. 6.**
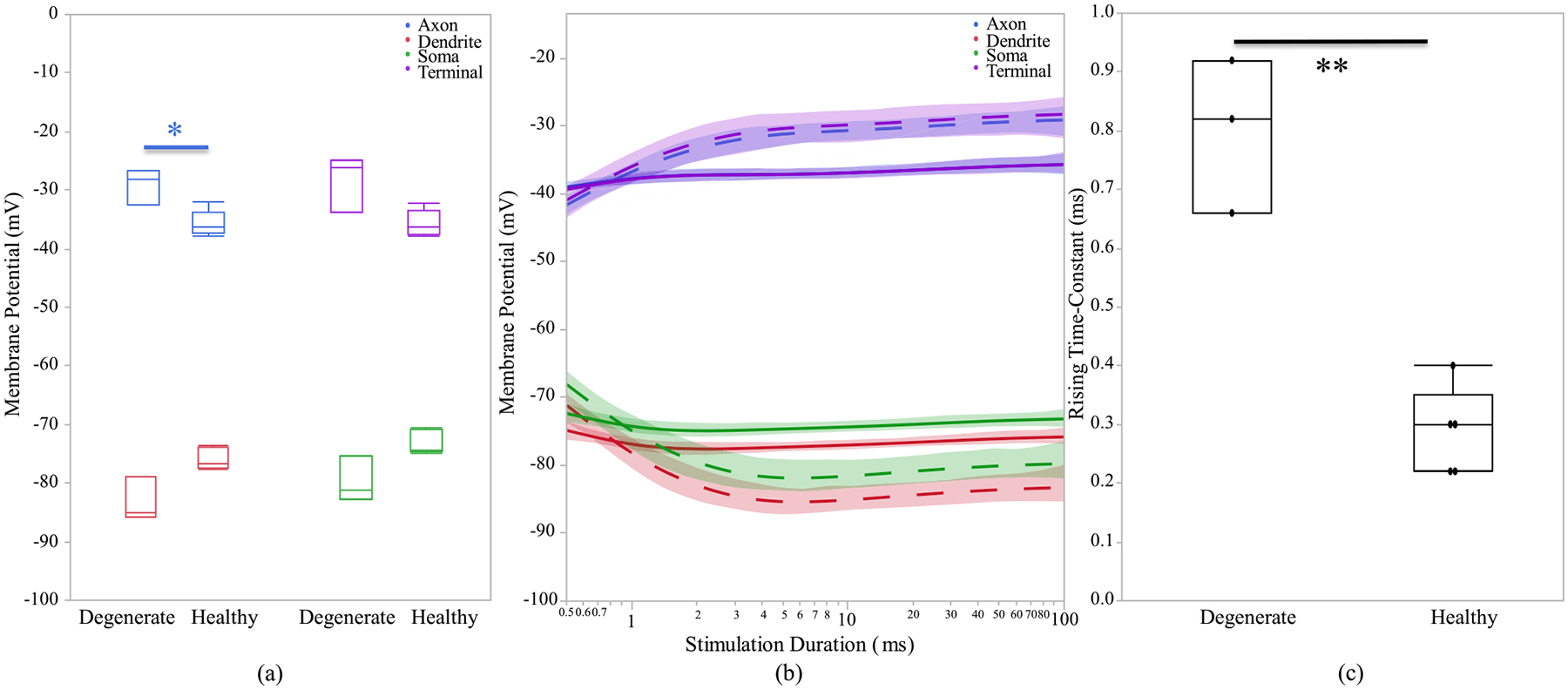
(a) Healthy and early-stage degenerated ON CBCs membrane potential response of different sections during 100 ms pulse duration, (b) membrane potential response with respect to stimulation duration. The healthy and degenerated cells are represented by solid and dashed line, respectively. (c) axon terminal membrane potential rising time-constant of 100 uA applied extracellular current. The * and ** are the t-test statistically significance level of ≤0.05 and ≤0.01, respectively.

**Fig. 7.**
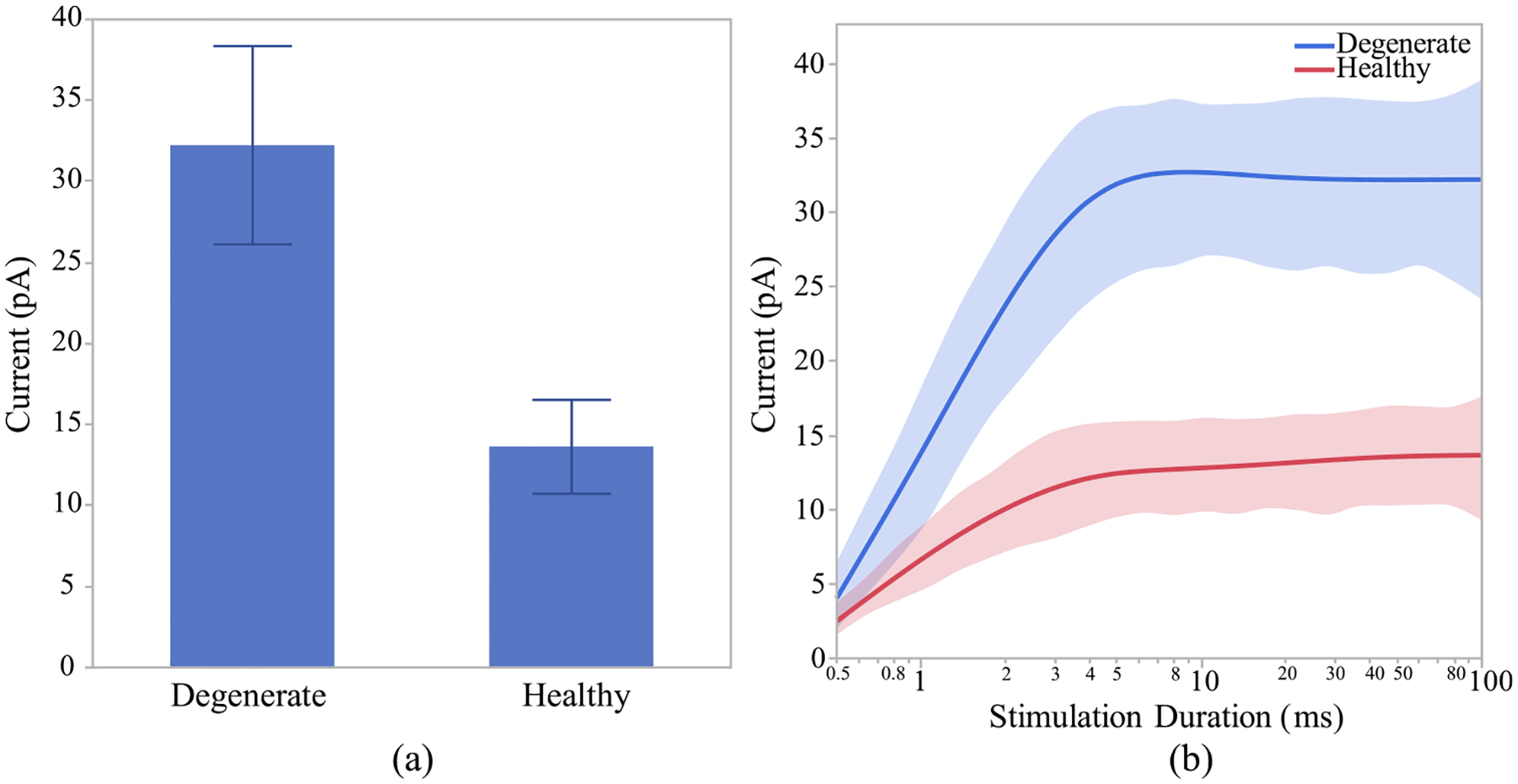
Healthy and early-stage degenerated ON CBC (a) average peak calcium current at the axon terminal during 100ms pulse duration and (b) average peak calcium current with respect to range of stimulation durations in logarithmic scale for 100 uA applied extracellular current.

After investigating the morphological differences of the ON and OFF CBCs of the healthy and early-stage degenerated retinas, we performed studies to analyze the membrane potential at all compartments of the cell and the calcium current response of the cells at axon terminals to extracellular current-controlled electrical stimulation. With the ON and OFF CBCs soma fixated at the INL, an increase in the membrane voltage, which corresponds to the depolarization of the cell, indicates that the healthy cells have significantly lower depolarization at axon sections than early-stage degenerated cells. Also, a decrease in the membrane potential, which corresponds to the hyperpolarization of the cell, indicated that the degenerated cells have insignificant lower membrane potential at soma and dendrite than the healthy cells. The healthy axon terminals also have lower depolarization than degenerated cells, however, the significance level is at 0.09,Fig. 6 (a). We also illustrate that the healthy ON CBCs reach peak membrane potential at shorter latencies than the early-stage degenerated ON CBCs, Fig. 6(b). Moreover, the results show that healthy ON CBCs have significantly shorter axon terminal membrane potential rising time-constant (0.29 ± 0.03 ms) than the degenerate cells (0.8 ± 0.07 ms), Fig. 6(c). The membrane potential rising time constant represents the time when the axon terminal membrane potential reaches 1/e ≈ 63.2% of the peak membrane potential value. Furthermore, the results from 100ms pulse duration Fig. 7(a) imply that the average peak calcium current at the axon terminal of healthy ON CBCs (13.64 ± 2.88 pA) is lower than the early-stage degenerated cells (32.24 ± 6.12 pA); however, the significance level is at 0.07. The plot in Fig. 7(b) illustrates the alteration of calcium current at the axon terminal of healthy ON CBCs continuously remaining lower than degenerate cells to increase pulse duration on a logarithmic scale. Rising time constant of L-type calcium current, *τ*_*I*_ −*T erm*, is not significantly different between the healthy (1.33 ± 0.16 ms) and degenerate (1.49 ± 0.30 ms) cells.

**Fig. 8.**
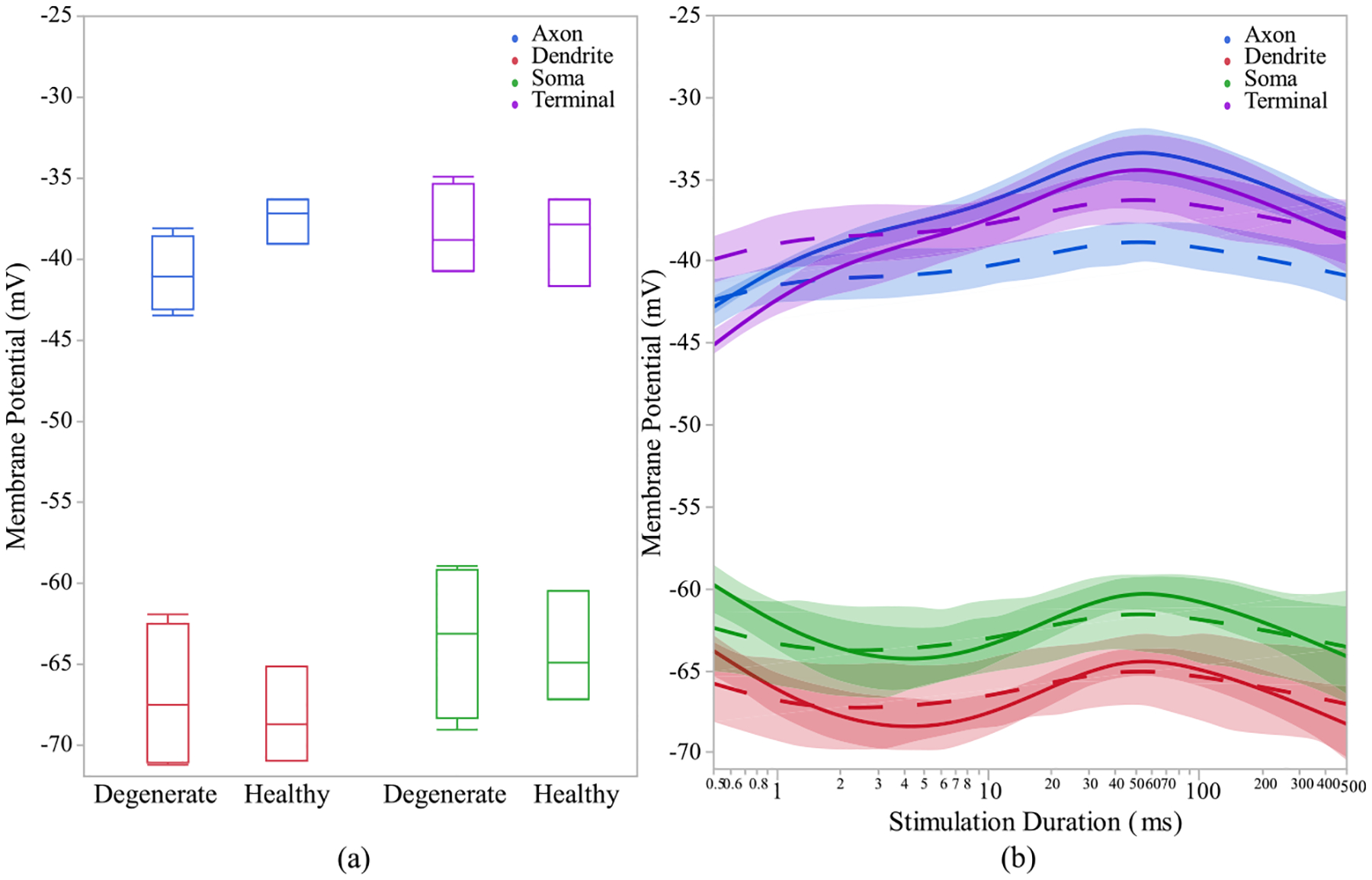
(a) Healthy and early-stage degenerated OFF CBCs membrane potential response of different sections during 500ms pulse duration, (b) membrane potential response with respect to log-scale stimulation duration of 100 uA applied extracellular current. The healthy and degenerated cells are represented by solid and dashed line, respectively.

**Fig. 9.**
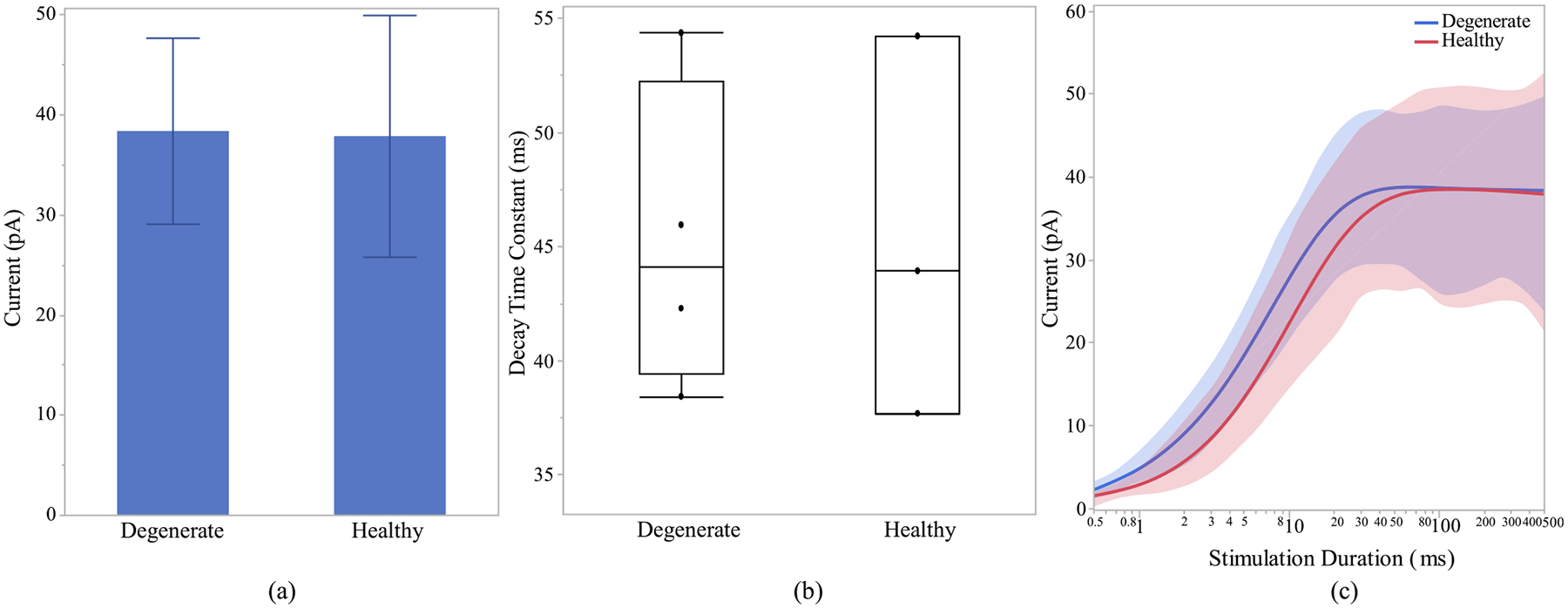
Healthy and early-stage degenerated OFF CBC (a) average peak calcium current at the axon terminal during 500ms pulse duration, (b) calcium current decay time-constant and (c) peak calcium current with respect to log-scale stimulation duration of 100 uA applied extracellular current.

The time for current’s peak value is also not significantly different between healthy and degenerate ON CBCs (data not shown). The tail current after the stimulation pulse at the upper right subfigure has been evaluated and it is significantly lower in healthy (14.74 ± 7.11 pA) compared with degenerated (39.55 ± 15.23 pA) cells with p < 0.05. In Fig. 8 (a), which shows the membrane potential of OFF CBCs of 100 μA extracellular current at a pulse duration of 500 ms, there is no discernible difference between the healthy and degenerate OFF CBCs. However, healthy OFF CBCs exhibit a lower depolarization (−45.35 ± 0.52 mV) compared to early-stage degenerated cells (−40 ± 1.15 mV) with p < 0.01, at the axon terminal section, as depicted in Fig. 8 (b). Fig. 9 (a) illustrates that the calcium current at the axon terminal of OFF CBCs is similar in both degenerated and healthy cells, the elevation is not statistically significant. Also, there is no significant difference in calcium current decay time constant at the axon terminal of the healthy OFF CBCs in comparison to early-stage degenerated cells, as shown in Fig. 9 (b). The range for healthy calcium current decay time-constant of T-type calcium channel agrees with previously reported values [53]. Also, there is no significant difference in calcium current decay time constant at the axon terminal of the healthy (45.29 ± 8.34 ms) OFF CBCs in comparison to early-stage degenerated (45.27 ± 6.80 ms) cells, as shown in Fig. 9 (b). The time to peak calcium current in healthy (37.25 ± 7.38 ms) and degenerate (29.43 ± 6.07 ms) OFF CBCs is not significantly different (data not shown). Furthermore, healthy cells need a longer pulse duration (25ms vs 50ms) to reach the peak calcium current at the axon terminal, as shown in Fig. 9 (c).

**Fig. 10.**
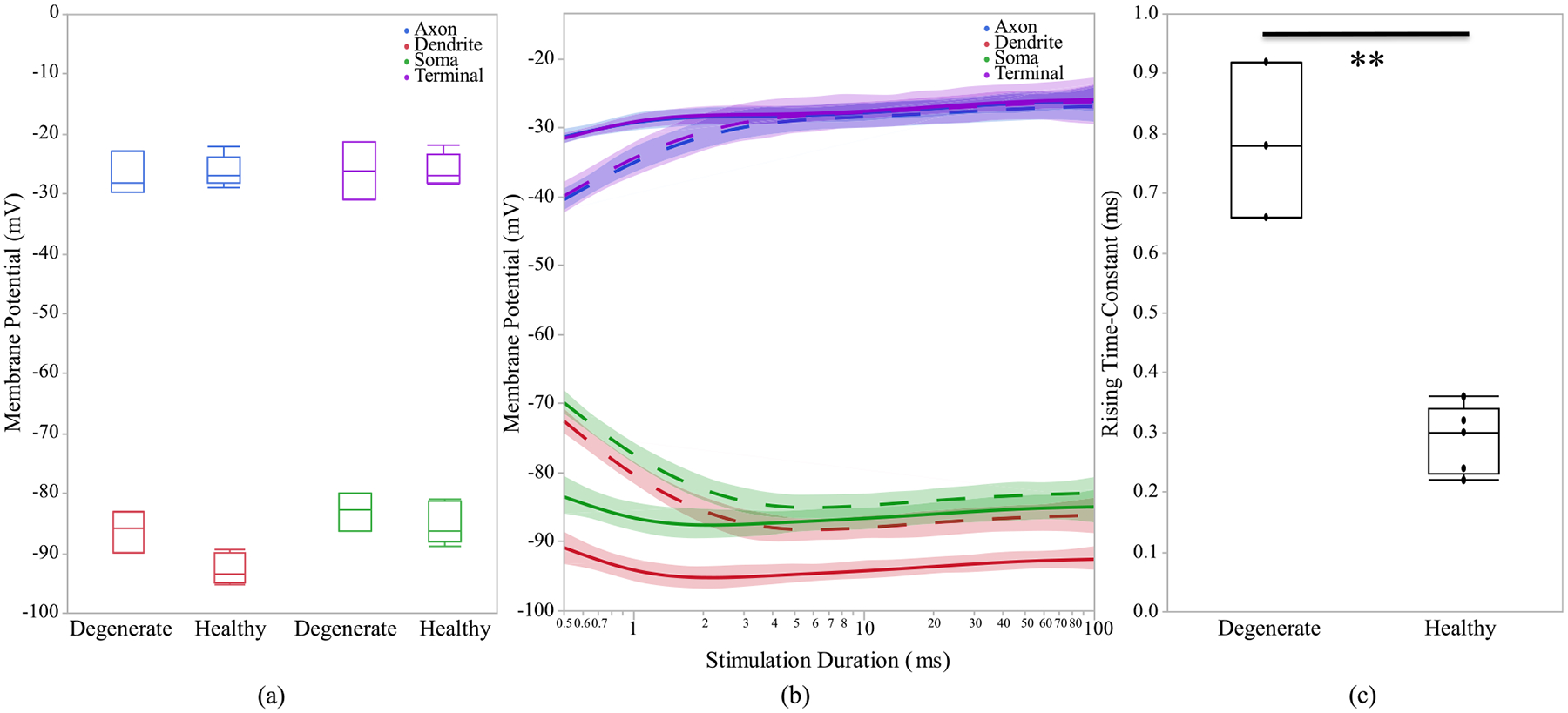
After adjusting the location of the cells based on the axon terminal position with respect to electrode: (a) Healthy and early-stage degenerated ON CBCs membrane potential response of different sections, (b) membrane potential response with respect to log-scale stimulation duration. The healthy and degenerated cells are represented by solid and dashed line, respectively. (c) synapse membrane potential rising time-constant of 100 uA applied. extracellular current.

**Fig. 11.**
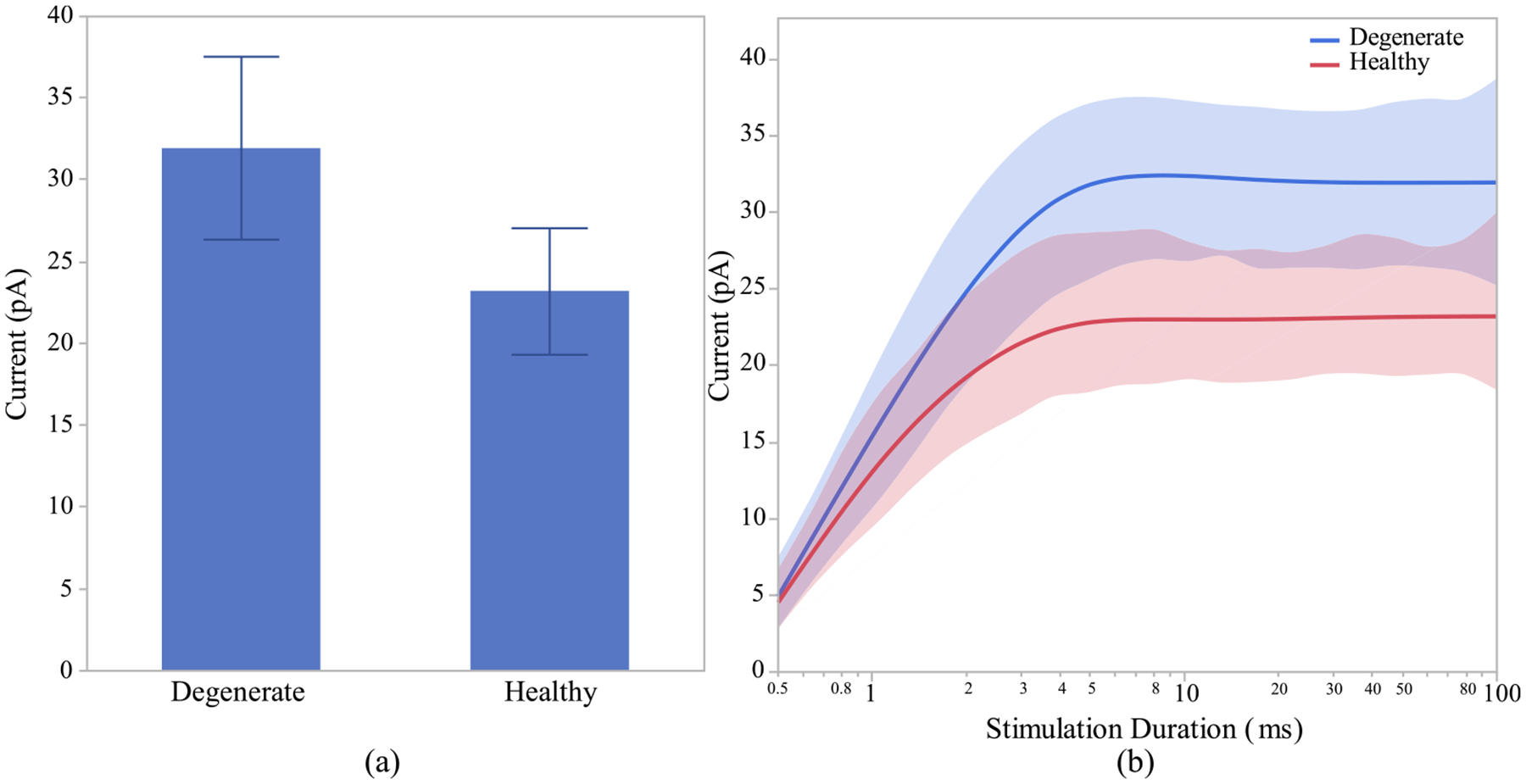
Healthy and early-stage degenerated ON CBC (a) average peak calcium current at the axon terminal for pulse duration of 100ms and (b) average peak calcium current with respect to log-scale stimulation duration of 100 uA applied extracellular current after adjusting the location of the cells based on the axon terminal position.

To evaluate the impact of relative cell positioning in the INL on signal flow, the cells have been adjusted so that all cells’ axon terminals have similar distance from the stimulating electrode. The results of cell location adjustment in the model indicate no significant difference between the cell’s membrane potential and calcium current responses of healthy and degenerate ON CBCs for pulse duration of 100 ms, as shown in Fig. 10 (a). However, we can still observe that the healthy ON CBCs require shorter stimulation pulse duration to reach peak membrane potential response, as shown in Fig. 10 (b). For a pulse duration of 0.5 ms, the membrane potential of the dendrite and soma in healthy ON CBCs is lower (−90.61 ± 1.69 mV and −83.25 ± 1.82 mV, respectively) compared to early-stage degenerate ON CBCs (−72.07 ± 1.2 mV and −69.43 ± 1.2 mV, respectively). The difference is statistically significant (p < 0.0001) for both measurements. Furthermore, the membrane potential of the axon and axon terminal of healthy ON CBCs at a pulse duration of 0.5 ms is significantly greater (−31.43 ± 0.7 mV and −31.61 ± 0.46 mV, respectively) compared to early-stage degenerate ON CBCs (−40.65 ± 1.09 mV and −40.16 ± 1.86 mV, respectively). The difference is statistically significant with p-values of < 0.01 and < 0.05 for the axon and axon terminal, respectively. The peak current at the axon terminals of the healthy ON CBCs is still lower than early-stage degenerated cells after the cell location adjustment but not significant, as shown in Fig. 11.Moreover, the effect of cell position modification on the response of membrane potential and calcium current in both healthy and degenerate OFF analysis indicates a significant impact (p < 0.05) only on the CBCs has been assessed, but not displayed. The statistical membrane potential of the axon terminal, particularly for short stimulation durations of less than 4ms. However, no noteworthy difference in calcium current response was observed.

## IV. DISCUSSION

We developed connectome-based computational models of ON and OFF CBCs in the healthy and early-stage degenerated retina. Our models of ON and OFF CBCs are based on neuronal morphologies extracted from RC1 and RPC1 connectomes. To the best of our knowledge, no electrophysiological studies have been conducted focusing on a morphologically realistic comparison of the healthy and degenerated CBCs. Previous studies have created a comprehensive model for spiking bipolar cells (BCs) in the magnocellular pathway of the primate retina, specifically targeting diffuse bipolar cells (DB4) [27], [60]. While active membrane properties have been confirmed in both ON and OFF BCs, previous BC models were either considered passive or only demonstrated L-type and T-type calcium channels at the cells’ axon terminals [24], [29], [30], [31], [32], [61]. The lack of sodium and potassium channels over the entire cell compartments affects generation of calcium action potentials.

The results of our study indicate that degenerated CBCs respond well to electrical stimulation, almost as good as healthy cells, suggesting the potential of electric stimulation in early disease stages. Furthermore, these models can be incorporated into larger network models to examine network level responses and used to study the selective activation of ON vs OFF CBCs. Additionally, the developed models can be refined to include more ion channels and replicate behaviors such as spiking.

In this study, ON and OFF CBCs illustrate fast depolarization at the axon and terminal sections and fast hyperpolarization at the soma and dendrite sections due to the extracellular current stimulation. This behavior has also been illustrated in wild-type and retinal degenerated mice for the INL cells [23]. Previous studies investigated the characteristics of calcium ion channels in goldfish and the neurotransmitter release is mediated by *Ca*^2+^ influx through the L-Type *Ca*^2+^ channels [62], [63]. There have been recent studies on mammalian bipolar cells and the effect of the L-Type and T-Type voltage-activated calcium currents in controlling the transmitter release [17], [32], [51]. Our results suggest that degenerate cells are less excitable than healthy cells based on the membrane potential rising time constant at the synapses. The connectome data has shown that degenerated cells in both ON and OFF CBCs have fewer axon terminals than healthy cells. Moreover, the results indicate that the calcium current is higher in the degenerate cells compared with the healthy ON CBCs. This implies that calcium channel concentration is higher since there is a direct relationship between calcium concentration and calcium influx. Additionally, our result for the decaying time constant for the transient T-type calcium channel is aligned with previous measurements [53]. Even with cell location adjustment in the model, the axon terminal calcium current is higher in degenerate cells, which consequently have higher calcium influx and intracellular concentration than healthy cells. Prior studies illustrated that the activation of *Ca*^2+^ channels at the axon terminal of bipolar cells could trigger transmitter release [54], [64], [65]. This could indicate that compensation for neurotransmitter release in CBCs is causing the degenerate cells to have higher intracellular voltage-gated calcium current with fewer terminals. Also, the results suggest that as the number of bifurcations and synapses increases the peak calcium current for ON CBCs decreases. However, the calcium current increases as the axon diameter and length increases. After adjusting the position of ON CBCs, the relationship between axon terminal peak calcium current still holds. Moreover, the degenerated ON CBCs have longer axons and larger axon diamete, which facilitates the calcium current rises in the remaining axon terminals. Again, this relation holds after adjusting the position of the cells.

For OFF CBC, the complex morphologically realistic models don’t show any significant differences in calcium current between health and degenerate cells. On possible reason for this could be that the axon path length and length of axons in OFF CBCs do not show significant differences between healthy and degenerated cells. These results withstand after adjusting the position of the cells based on their terminal position with respect to the electrode. Previous studies [6], [66], [67], [68] have shown that the axon bifurcations and synapses of degenerating cone bipolar cells decrease due to photoreceptor degeneration which matches our finding in both ON and OFF CBCs morphological analysis. They also observed a no significant difference in axon diameter due to degeneration which conform with our finding. Therefore, the complex morphologically realistic models facilitate the better assessment of the membrane potential and calcium current response distinctions between healthy and degenerated ON and OFF CBCs. These results help further our understanding of current flow through these important retinal cells, but network-level stimulations are required to explore the implication(s) of altered CBC calcium channels and membrane potentials between healthy and degenerate networks. By applying the cathodic monophasic pulse current stimulation, the results indicate that both ON and OFF CBCs are depolarized after stimulation. With the present stimulation pulse type selective or preferential activation of the ON and OFF CBCs has not been observed. To achieve selective activation of either type of CBCs, in the future, we would investigate how other stimulation types and durations help to reach this goal. A previous study [69] illustrated that bipolar cells are differentiated based on soma position in the INL. Hence, the response of cells whose soma is anchored in the INL provides a more accurate representation of the actual positioning of the cells. To assess the axonal terminal response of ON and OFF CBCs under identical conditions, we also modified their terminal distance from the stimulating electrode to determine if this affects their calcium channel response at the axonal terminal. However, there is no significant impact on the membrane potential and calcium current responses in both ON and OFF CBCs. Our computational modeling is also compatible with other types of retinal prosthetics including subretinal implants. In epiretinal prostheses the electrode is closer to the axon terminals of BCs, however, in subretinal prostheses, the stimulating electrode is closer to the dendrites of BCs. Therefore, the electrode position adjustment changes the polarity of the stimulus waveforms [40], [60]. Also, for epiretinal stimulation, the voltage gradient is closer to the ganglion cell side. This facilitates preferential stimulation of ganglion cells. For subretinal stimulation, the maximal gradient would be closer to the bipolar cells.

This paper focuses exclusively on examining the involvement of L-type and T-type calcium channels specifically in the axon terminals of both healthy and degenerated ON and OFF CBCs using a morphologically realistic approach. However, further studies are needed to explore the impact of ion channels distribution in other parts of the cells. The results of our study indicate that degenerated CBCs respond well to electrical stimulation, almost as good as healthy cells, suggesting the potential of electric stimulation in early disease stages. Furthermore, these models can be incorporated into larger network models to examine network level responses and used to study the selective activation of ON vs OFF CBCs. Additionally, the developed models can be refined to include more ion channels and replicate behaviors such as spiking.

In the future, we plan to incorporate inner retinal layer cells (amacrine cells and ganglion cells) into our computational models and investigate how these observed RPC1 CBCs impact the response of the degenerated retinal network. These models will help us understand the changes in the retinal network with the progression of the disease and potentially assist in designing better stimulation strategies. To simplify the analysis, we have restricted the CBC comparison to a single class when sufficient cells were available. Additionally, we will examine each of these distinct classes separately to capture the cellular response to electrical stimulation. To simplify these computational models, we have assumed that the biophysics of degenerated cells is equivalent to that of healthy tissue since we do not possess model parameters that are specific to degenerated RPC1 ON and OFF CBCs pathoconnectome experimental recordings found in the literature.

There are a few limitations to this work. First, the morphology and synaptology of the dendrites for healthy ON and OFF CBCs could not be directly extracted from the RC1 connectome. As we have utilized the dendritic information extracted from a corresponding ON and OFF CBC from RPC1 to complete the cell morphology of RC1 ON and OFF CBCs, morphological comparisons between RC1 and RPC1 cells were therefore restricted to the soma and axonal compartments. Second, the cells were extracted from two animals, one healthy and one in the early phases of photoreceptor degeneration. As the process of extracting and annotating morphology from the connectome is incredibly costly and time-consuming, only a limited number of cells have been simulated. However, it is anticipated that as the database becomes more comprehensive in the future, a greater number of cells will be simulated. Moreover, mammalian CBCs are a heterogenous population comprised of multiple classes that differ in their morphology, synaptology, and network function [69], [70], [71], [72], [73]. Lastly, several aspects of cell morphology and synaptology can differ as a function of location [74], [75], [76], [77]. As the RPC1 connectome was derived from tissue positioned closer to the visual streak of the rabbit than the tissue obtained from the healthy adult rabbit retina for RC1, the relative contributions of tissue location and degeneration to the observed morphological differences remain unclear. However, given the clear effect of these morphological differences exerted on cell behavior in the simulations, how such differences may be compensated for in the network architecture across the healthy retina would also be of significant future interest.

## V. CONCLUSION

Understanding the changes in the degenerated retinal network promises to help understand the underlying pathogenic processes and the changes in network-level response. This study investigated the effect of the calcium current at the axon terminal of morphologically realistic early-stage degenerated and healthy ON and OFF CBCs. The benefits of having morphologically realistic cells improve understanding of the mechanism behind the active channels available at axon terminals and help optimize the stimulation pattern and duration to activate the ON and OFF CBCs in a degeneration state. Within this context, our predictive modeling framework aims to facilitate the development of more efficient stimulation strategies in retinal prosthetic systems. Using this framework, we demonstrated that regardless of the position of the OFF CBCs in the retina model, there is no difference between the membrane potential of healthy and degenerate cells when electrically stimulated. However, the membrane potential of healthy and early-stage degenerated ON CBC differs based on the cell’s position in the model. Specifically, the degenerated ON CBCs would have higher depolarization values at the axon and terminals sections and higher hyperpolarization values at the soma and dendrite sections compared with the healthy cells when the synapses are positioned in the inner IPL. However, this significant difference is not noticeable when we place the synapses closer to the electrode. Although the membrane potential of ON and OFF CBCs may differ based on the cell’s position in the model, the calcium current and intracellular concentration at the axon terminals are still significantly higher in degenerate cells than in healthy cells, even after adjustment of the position of the cell.

Our results illustrate that the ionic calcium channels at the axon terminals of the cells have a higher concentration and current in degenerated cells independently of the cell’s position; instead, the main factor driving this change is the morphological axonal branching and terminals differences. This indicates that fewer axon terminals at degenerate cells are causing higher currents to go through the synapses compared with healthy cells. This could be CBC degeneration compensatory mechanism as the prior studies suggest that activation of *Ca*^2+^ channels at the axon terminal of bipolar cells could trigger transmitter release.

The computational models developed in this work will serve as a building block as we move towards modeling richer retinal networks and analyzing the network-level impacts of morphological changes and new abnormal axon terminals observed in the degenerated retina. Evaluating the morphological and structural differences between healthy and degenerated retina would assist in adjusting the stimulation strategies used in current prosthetic systems.
